# Breastfeeding results in better hearing in newborns compared to bottle-feeding

**Published:** 2020-08-29

**Authors:** Jose Miguel Sequi-Canet, Jose Miguel Sequi-Sabater, Jose Ignacio Collar-Castillo, Nelson Orta-Sibu

**Affiliations:** ^1^Department of Pediatrics, Hospital Universitario Francesc de Borja, Gandia, Spain; ^2^Department of Rheumatology, Hospital Universitario Reina Sofia, Córdoba, Spain; ^3^Visiting Professor, Hospital Universitario Francesc de Borja, Gandia, Spain

**Keywords:** breastfeeding, otoacoustic emissions, newborn hearing screening

## Abstract

**Background and Aim:**

Transient evoked otoacoustic emissions (TEOAEs) are a validated technique in newborn hearing screening that is regularly used in many countries. It reflects normal hearing or at least no more than 30 dB HL hearing loss. Breastfeeding has many advantages and some studies have demonstrated that it prevents otitis media by means of opening the Eustachian tube and clearing mucus in the middle ear which is perhaps also combined with immunological effects. A few studies have related how newborn feeding can vary the pass rate to TEOAE. The goal of this study was to investigate the relationship between newborn feeding and TEOAE newborn hearing screening results.

**Methods:**

Data were retrospectively collected from healthy vaginally delivered newborns of gestational age >37 weeks and body weight > 2.5 kg at the maternity ward. Newborn feeding history was compared with the pass rate to TEOAE performed within the 1^st^ 48 h of life.

**Results:**

The study group included 12,866 newborns. In this group, significant differences were found based on the feeding method (breastfeeding was found to be better than formula, *P*<0.0001).

**Conclusions:**

Breastfeeding improves newborn hearing screening results with TEOAE.

**Relevance for Patients:**

Lies in the fact that breastfed children respond better to the test and need to repeat it fewer times, avoiding problems such as loss to follow-up and additional work.

## 1. Introduction

Universal newborn hearing screening is routinely performed because only 50% of babies born with hearing loss carry a hearing loss risk factor. Early detection leads to an efficient treatment of the affected neonates, resulting in a better final prognosis [1-3].

There are several techniques used in newborn hearing screening. Otoacoustic emissions (OAEs) are low-level acoustic signals generated by the cochlea and passed through the middle ear into the external ear canal. OAEs are an objective indication of normal cochlear function, unlike pure-tone audiometry; OAE-based screening does not require any behavioral cooperation from the testee which makes it a very good screening method for infants. OAEs occur in nearly all ears with normal hearing and middle ear function. Transient evoked otoacoustic emission (TEOAE) testing is one of the most frequently used techniques due to its accuracy, simplicity, speed, and low cost, as described in diverse studies [2,4-6].

Researchers have compared the sensitivity of evoked OAE testing with pure-tone audiometry and concluded that OAE testing is more sensitive in detecting the early onset of cochlear pathologies before a change in hearing thresholds occurs [7].

A major drawback of TEOAE testing as a screening technique for newborns relates to the middle ear status, which can severely affect its pass rate. In addition, the presence of debris and vermix in the external ear meatus of the newborn can result in false-positive screenings. This factor can lead to an overestimation of the actual hearing loss failure rate.

Another crucial factor is the newborn’s age at the moment of testing. Data strongly suggest that the prime testing window is beyond 24-48 h of life, as fluid in the middle ear and in the external meatus is normally significantly reduced on the 2^nd^ day of life. For this reason, the TEOAE test is done as near as possible to discharge. The average stay in our hospital for mothers following a vaginal delivery is 48 h, and for cesarean section, it is more than 72 h, this allowed for a successful hearing screening implementation program [2,7].

Many sources, including the Joint Committee on Infant Hearing and the Commission for the Early Detection of Hearing Loss (CODEPEH) based in Spain, define well-known hearing loss risk factors [8-10]. However, some studies have demonstrated the existence of other epidemiological factors that modify TEOAE test results [11]. One of such factors appears to be the feeding type (breastfed newborns seem to have a better response to TEOAE), which seems to modify the pass rate to hearing screening test as described in various studies, but for which a clear explanation has not been provided. Breastfeeding has many advantages and some studies in infants have demonstrated that it prevents otitis media by means of opening the Eustachian tube and clearing mucus in the middle ear which is perhaps also combined with immunological effects, but in newborns, the real effect of breastfeeding on response and pass rate to the TEOAE screening test is yet to be confirmed [11-15].

The objective of this study is to answer the question of whether newborn feeding can truly influence the newborn TEOAE screening results and modify the TEOAE pass rate.

## 2. Patients and Methods

Significant differences in TEOAE amplitudes between groups can alter the pass rate of screening tests; therefore, the goal of this study was to compare newborn feeding history with influences on the pass rate to the TEOAE test as a method for newborn hearing screening during the first 48 h of life with the hypothesis that breastfeeding might help us obtain PASS results on the first couple of days since the Eustachian tube might take longer to open and provide middle ear aeration in bottle-fed newborns.

Data were collected between 2000 and 2019 from all healthy newborns without any known hearing loss risk factor in the maternity ward. This retrospective study was approved by the ethical committee of this hospital on July 15, 2019 with code 12/2019.

### 2.1. Exclusion criteria

The focus of the study was limited to healthy newborns without any syndrome or known disease. In addition, newborns with Apgar lower than 7 at 5 min were excluded from the study. Only vaginally delivered newborns were included because timing is a crucial factor in response, and neonates delivered by cesarean section stay in the hospital 72 h, so the TEOAE test is done around this age.

To eliminate other possible confounding factors [16], only newborns older than 37 gestational weeks with a birth weight greater than 2.5 kg were included in the study. There were no differences between feeding groups based on gender.

### 2.2. Protocol

The bilateral TEOAE screening was performed as close as possible to 48 h of life. Sometimes an initial TEOAE test near discharge at 48 h was done, even though the baby was a little fussy because the baby and the screener were both available at that moment. However, if the baby “failed,” it is assumed that it was because the baby was fussy and another test was done a few hours later, immediately before discharge; most of these babies passed the test. Thus, in this case, the second test was considered a more valid result and was the one included in this study. If the baby passed, no more testing was done.

All nurses performed the screening, on every shift, every day of the week, based on availability. The screening was performed in the newborn room with as little background noise as possible after parental verbal consent was obtained. Testing took place after feeding time to ensure the newborn was calm and also to avoid inherent noises related to feeding. No sedation was administered.

### 2.3. Techniques

The TEOAEs were recorded with an ECHOCHECK OAE Screener based on the ILO88 (Otodynamics, Hatfield, U.K.) system and connected to the ILO ECP neonatal probe. This emits a standard click-type non-linear stimulus of 1 ms duration. The intensity of which is 84 ± 3 dB SPL (sound pressure level) 80 times/s receives and averages the responses produced by the cochlea to OAEs from 1-4 kHz with a primary response band of 1.6-3.2 kHz., the 1.6 kHz frequency is filtered to avoid noise contamination.

The device is small and portable. Its settings automatically adapt to the size of the external auditory canal. It has luminous signals that confirm that the stimulus is reaching the ear correctly and that the noise level is admissible for the test (<47.3 dB SPL on average, although in certain frequencies may be higher). “Pass” results indicate that there are TEOAEs. A normal result (pass) requires a signal/noise level response above 6 dB with a minimum of 512 valid responses for at least 5 s. The duration of the test usually oscillates between 45 s and a maximum of 5 min.

A newborn with normal bilateral response was accepted as a pass; otherwise, it was deemed a fail [17].

### 2.4. Statistical analysis

The dependent variable is the TEOAE result before discharge at 48 h of life (pass/refer).

The independent variable is the newborn’s feeding type registered in maternal history (breast/formula).

Following frequency analysis of the variables, a univariate analysis was completed between the TEOAE results and the study variables with the Chi-squared test and risk estimate with odds ratio.

Statistical analyses were only conducted on patients that had data available for either of the study variables (feeding vs. TEOAE results).

The significance level was established at p < 0.05. The data were analyzed using Excel 2016 and SPSS version 20.

## 3. Results

Table 1 shows that breastfeeding was the feeding type in the majority of newborns (73%). In spite of this, there are enough cases in both groups.

**Table 1:** Newborn feeding type.

Feeding method		Frequency	Valid percent
	Formula	3469	27.0
Feeding	Breast	9397	73.0
	Total	12866	100.0
Missing		5	
Total		12871	

Table 2 shows that related to feeding type, there is a significantly (p<0.0001) higher percentage of fails to TEOAE found in formula-fed (9.7%) versus breastfed newborns (7%) and also related to TEOAE results, there is a higher percentage of fails versus pass in formula-fed newborns (33.8% vs. 26.3%) than in breastfed (66.2% vs. 73.7%). The odds ratio of failing for formula-fed newborns was 1434 (1249–1648).

**Table 2:** Crosstab feeding versus TEOAE results.

Feeding method			TEOAE		Total	

			TEOAE FAIL	TEOAE PASS		
Feeding	Formula	Count	331		3083	3414
		% within feeding	9,7%		90,3%	100,0%
		% within TEOAE	33,8%		26,3%	26,8%
	Breast	Count	648		8658	9306
		% within feeding	7,0%		93,0%	100,0%
		% within TEOAE	66,2%		73,7%	73,2%
Total		Count	979		11741	12720
		% within feeding	7,7%		92,3%	100,0%
		% within TEOAE	100,0%		100,0%	100,0%
**Pearson Chi-Square**			**Value**	**df**	**Asymp. Sig.(two-sided)**	
		
			26,244	1	0.0001	

**Risk Estimate**		**Value**	**95% Confidence interval**			

			Lower		Upper	

Odds ratio for feeding (formula/breast)		1.344	1.249		1.648	

## 4. Discussion

The percentage of breastfed babies in our study (which does not include 3% mixed feeding) was 73.16%. Although this appears not to be very high, the large study period (19 years) must be considered. Over the last years, education on breastfeeding has raised figures to around 75%-80%, which is more in line with current trends. The advantage being that the comparison group of formula-fed babies was therefore sufficiently large.

Testing took place following feeding time, because adequate TEOAE response needs the baby to be calm to avoid as much noise as possible and also because it seems that babies that are bottle-fed make more noises (gulping and gasping), and therefore, results would be more difficult to obtain compared to breastfeeding babies.

There are some studies that show a better pass rate to TEOAE screening in newborns fed with breast milk. In a former study, in a different group of newborns, about diverse perinatal factors influencing TEOAE results, we have informed in a preliminary way about a significant difference in response between breast and formula-fed newborns [11].

The objective of this study is to analyze pass rate to TEOAE newborn hearing screening depending on feeding type in a selected group of healthy newborns, which were vaginally delivered, to term and at a normal weight, without hearing risk factors, and as close as possible to 48 h of life before discharge from the maternity ward to avoid some confounding factors cited in other studies [12-16].

An age approaching 48 h was selected because it is clear that before 24 h of life, the fail rate is much higher than later [18]. That is the reason why newborns that were delivered by cesarean section were excluded from the study since they are usually tested on the 3^rd^ day, and it is known that TEOAE’s results are more accurate beyond the 3^rd^ day.

Healthy term babies in the maternity ward were selected because some studies show that immaturity, low birthweight, and therapy in the intensive care ward are important factors that disturb the OAE [19] and compared to term infants, late preterm infants (35-37 weeks) had 2-fold higher rates of failure on 1^st^ OAE (up to 42 h of life) and needed repeated hearing tests [20].

Our results show that there are very significant differences in TEOAE results depending on the type of feeding. There was a significantly lower proportion of failing TEOAE results in the breastfed group compared with the formula-fed one (7% vs. 9.7%; p<0.0001) and an odds ratio (OR) of 1.43 (CI 1.25-1.65) for failing the test was calculated in the formula group.

If we look at all the babies that have TEOAE done, you realize that formula-fed represents 26.8% of all TEOAE, and in this special group, one can see that there are more fails (33.8%) than pass (26.3%). The contrary occurs with breastfed babies, they represent 73.2% of all TEOAE done and the percentage of fails (66.2%) is lower than the pass (73.7%).

These results agree with a higher rate of hearing loss in formula-fed infants, as shown recently by Van Kerschaver [12] in a study with a population of 103,835 term newborns in Flanders, Belgium. These newborns were tested by a universal neonatal hearing screening (UNHS) program. Using automated auditory brainstem responses (AABR), they concluded that there was a significant association between breastfeeding and the prevalence of congenital hearing impairment (CHI) failing AABR. This effect remained after adjusting for the origin of the mother and other factors. Breastfed newborns were less likely to have CHI than their bottle-fed counterparts. Although feeding type is linked to education level, origin of the mother, environmental factors, as well as to poverty and smoking habits, logistic regression analysis has shown that feeding type appears as an independent variable, which contributes to the prevalence of CHI. This study remains inconclusive on the exact mechanism of the complex relationship of feeding type with CHI. Since, in this country, poor people are less likely to breastfeed, they hypothesize that breastfeeding, through the path of poverty, is linked to CHI. This could be an explanation for our results, but we think that there is a better explanation based on other physiologic reasons because there is no exact mechanism that may link congenital hearing impairment to breastfeeding since breastfeeding on its own can only be potentially considered as a postnatal cause. Furthermore, it remains unclear why poor people are less likely to breastfeed (is it due to poor health?) since one can only make the assumption that poor people are less likely to be able to bottle-feed their newborns due to a high cost.

The rationale for this difference is probably better explained based on middle ear status since diverse studies have demonstrated that breastfeeding alone can be considered a protection factor against middle ear changes. For example, Garcia [13] published an article where Otoacoustic emissions (OAE) were carried out in 60 infants between zero and 4 months old. The breastfed infants had a higher occasion of normal tympanometries and normal otorhinolaryngological assessment enabling better OAEs, with statistically significant differences.

The mechanism for these differences lies in the theory that an earlier opening of the Eustachian tube and/or a better middle ear clearance due to the position of the baby or the suction movements for breastfeeding can explain this.

There has also been the suggestion that it is the method of feeding (bottle vs. breast) that creates an increased risk of otitis media (OM), regardless of whether the bottled milk is formula or expressed breastmilk. The position of the infant during feeding (supine or semi-upright) has been proposed as an explanation.

Boone [21] showed that 1 month of feeding at the breast was associated with 4% reduced odds of ever having otitis media and for infants fed at the breast for 6 months, the reduced odds were 17%. Among infants who were bottle-fed with expressed milk in the first 6 months postpartum, the odds of experiencing otitis media increased by approximately 14% for infants fed for 1 month and by 115% for infants fed for 6 months. This finding suggests that the feeding mode rather than the substance fed underlies the differences in the risk of otitis media [21].

In addition, Tully *et al*. [22] reported a 59.6% rate of abnormal tympanograms following supine bottle-feeding compared to a 15.0% rate of abnormal tympanograms in infants fed in a semi-upright position, regardless of the contents of the bottle. They argued that supine bottle-feeding results in aspiration of milk into the middle ear cavity resulting in blockages that may be linked to an increased incidence of OM. However, Rosenfeld [23] has argued that while supine feeding may result in abnormal tympanograms for infants, these infants did not have a history of OM, and therefore, the effect of supine feeding on children prone to OM has not yet been established.

It has also been established that the mechanics of infant sucking for bottle-fed or mixed-fed babies are different from that of breast-fed babies, with fewer sucks and longer pauses observed for bottle-fed babies [24]. Infant jaw movement facilitates opening and closing of the Eustachian tube [25,26], and the reduced sucking movements in bottle-fed infants may result in less ventilation of, or reduced clearance of fluid from, the middle ear. This reduced ventilation of the middle ear in bottle-fed infants may be another mechanism for increased risk of OM in this group.

There are also physiological mechanisms that could explain the association between breastfeeding and reduced risk of OM. Strong negative pressure is generated by breastfeeding, in contrast to bottle-feeding. Suck, swallow, and breathing patterns are also different from bottle-fed infants [27,28].

All this can explain why breastfed newborns show a better response to TEOAE newborn hearing screening since they will have a better middle ear status from the beginning and, as discussed above, this is correlated with the good evidence from systematic reviews and meta-analysis showing a protective effect of breastfeeding on the risk of OM in the first 2 years of life [28]. In addition to the biochemical components in human milk, breastfeeding clearly protects from otitis media as concluded in a study by Brennan-Jones [29] who found from a study of 1344 children, which were part of a 6-year cohort follow-up and were given ear and hearing assessments, a positive association between formula feeding and otitis media in early childhood showing a protective effect of breastfeeding.

## 5. Study Limitations

The Echocheck screener results do not provide actual TEOAE response amplitude values. The TEOAE test without normal results indicates a hearing loss greater than 30 dB HL. Additional studies using actual response amplitude data are needed to consider the amount of difference in response.

The non-linear protocol used in the current study is the most common method used to record TEOAEs [29]. This method uses three clicks of one polarity with a subsequent single click with 3 times the amplitude and opposite polarity. The test can detect cochlear responses in the presence of linear artifacts related to the clicks. However, part of the actual OAE recording is eliminated as all linear components of the response are removed. Therefore, non-linear measurement may not be able to detect the OAE response completely; this process results in a low signal-to-noise ratio of TEOAEs in general. Perhaps, it is necessary that the linear measurement of TEOAEs should also be recorded in addition to using a non-linear protocol to clarify this issue in future research.

The Echocheck screener explores a frequency range from 0 to 4 kHz. Further studies are required to determine if there is any effect in some of the frequencies outside of this range, such as differences in higher frequencies that cannot be detected with this device.

Given that healthy newborns were examined for this study, it remains unknown if formula feeding increases the susceptibility to other neonatal hearing loss factors. In addition, perhaps there are other unknown perinatal factors that can vary response in formula-fed newborns. More studies in this area are needed.

## 6. Conclusion

Breastfeeding is an important factor related to a normal response in OAEs test. It may improve the final results of newborn hearing screening reducing the number of neonates who need to be rescheduled for a repeat test, as well as the associated anxiety and the possibility of losing patients during follow-up. These are major problems in neonatal hearing screening. This is another good reason to encourage newborn breastfeeding.
